# Lidar-derived digital surface model of Stromboli island updated to August 2023

**DOI:** 10.1038/s41597-025-04856-6

**Published:** 2025-03-28

**Authors:** Marina Bisson, Roberto Gianardi, Riccardo Civico, Paolo Madonia, Tullio Ricci, Claudia Spinetti

**Affiliations:** 1 Istituto Nazionale di Geofisica e Vulcanologia, Sezione Pisa, Pisa, Italy; 2 Dipartimento di Scienze della Terra, Università di Pisa, Pisa, Italy; 3 Istituto Nazionale di Geofisica e Vulcanologia, Sezione Roma 1, Roma, Italy; 4 Istituto Nazionale di Geofisica e Vulcanologia, Osservatorio Etneo, Catania, Italy; 5 Istituto Nazionale di Geofisica e Vulcanologia, Osservatorio Nazionale Terremoti, Roma, Italy

**Keywords:** Geomorphology, Volcanology

## Abstract

Digital surface models reproduce the 3D topography of a territory at different spatial resolutions depending on the acquisition technique of source data. In active and densely populated volcanic areas, updated digital topographies are fundamental for mapping and quantifying the morphological changes generated by the eruptive events and play a key role in modelling volcanic phenomena and related hazards. This work presents the high-resolution Digital Surface Model of Stromboli Island, Italy, updated to 4^th^ August 2023. The model, obtained by elaborating more than 109 × 10^6^ Airborne Lidar points (x,y,z), reconstructs the volcano’s surface through an elevation matrix at a spatial resolution of 50 cm, reproducing both natural and anthropic elements. The model has been validated by using Ground Control Points and the vertical accuracy results in 8 cm. Nowadays, this model represents the most updated and accurate digital 3D topography of the entire island and, for this reason, can be considered a relevant data not only for multi-temporal morphological and volcanological analyses but also for hazard assessment studies.

## Background & Summary

Active volcanoes continuously modify their morphology in response to eruptive activity and associated secondary phenomena such as landslides and wildfires.The reconstruction of the topography before and immediately after a volcanic event is essential for deriving qualitative and quantitative data on landscape changes and for volcanological purposes.

The surface of active volcanoes is generally reconstructed elaborating remote sensing data with different techniques. The main techniques and related data are: (i) Structure from Motion (SfM) applied to images acquired from Unoccupied Aircraft Systems (UAS) or crewed overflights; (ii) triangulation of elevation points acquired through Airborne Laser Scanning (ALS) and Terrestrial Laser Scanning (TLS); (iii) Stereo Photogrammetry applied to satellite imagery.

The above mentioned methodologies allow the reconstruction of topographies with a wide range of spatial coverage, spatiotemporal resolution and accuracy. The choice of the methodology mainly depends on two factors: the purpose for which the reconstructed topography is used and the cost-benefit assessment. Moreover, regarding geomorphological studies, it is also important to define the scale of analysis to select the most suitable methodology. Usually, for detailed geomorphological studies of active volcanoes, the mostly used methodologies involve data acquired by UASs, ALS and TLS^1–7^.

**Fig. 1:**
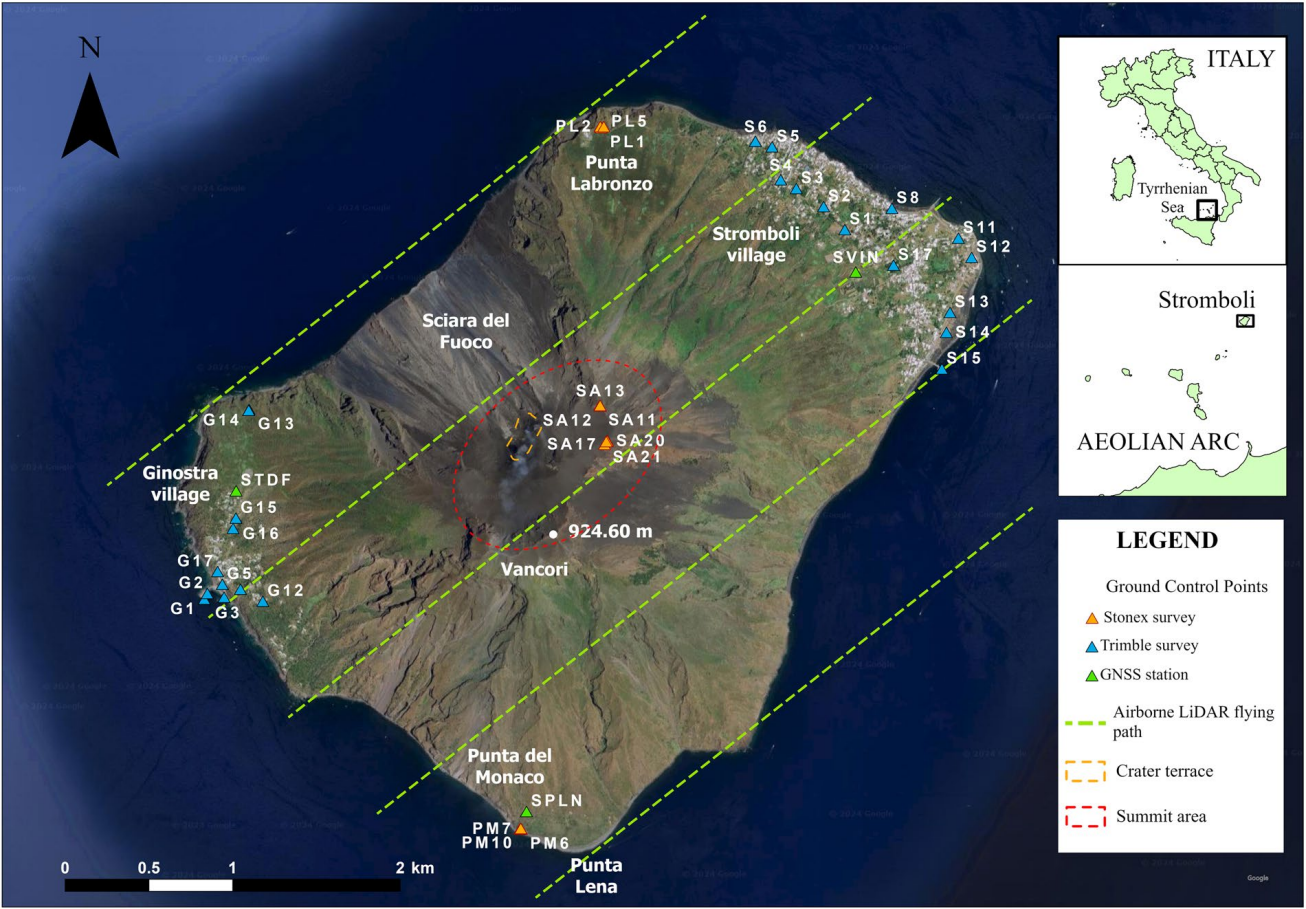
Stromboli Island map. The Ground Control Points (GCPs) locations and the Airborne Lidar flight lines are visualized on a Google Earth image of the island. The insets at the top right of the figure show the location of Stromboli Island.

Stromboli Island is the northernmost volcano of the Aeolian Archipelago, located in the southern Tyrrhenian Sea, Italy (Fig. 1). It extends for about 12.58 km^2^ and represents the subaerial portion of a volcanic edifice, which rises ~3000 m above the seafloor^8^. At Stromboli Island, the volcanic activity occurs within the summit area, specifically in the crater terrace positioned at ~750 m a.s.l., and along the Sciara del Fuoco (Fig. 1). The volcano is characterised by an ordinary activity consisting of mild strombolian explosions (5–30 explosions per hour), ejecting lapilli and bombs up to a few hundred metres above the vent, and a few tens of metres beyond the edges of the crater terrace. These ejecta frequently roll down along the Sciara del Fuoco, a horseshoe-shaped depression involving most of the western flank. The mild activity is occasionally interrupted by energetic, impulsive explosions, lasting from tens of seconds to a few minutes, named major explosions and paroxysms, according to the magnitude and intensity of the event^9,10^. Concerning the paroxysmal activity, the most recent events occurred on July 11^th^ 2024, August 28^th^ 2019 and July 3^th^ 2019. The two 2019 paroxysmal eruptions strongly changed the crater terrace morphology^11^, emitting a great amount of material, consisting of ash, lapilli, bombs and spatter clasts, which fell down the flanks and the summit of the volcano. The whole erupted mass is estimated in the order of 10^8^ kg, according to Bisson *et al*.^12^ and references therein. Furthermore, the paroxysms generated not only pyroclastic flows into the Sciara del Fuoco, triggering small tsunamis at their sea entrance, but they also produced at least two gravity-induced flows^13,14^ and triggered wildfires, especially in the south-east and south-west sector of the island^15,16^.

The period after the 2019 paroxysms, between August 28^th^ 2019 and the end of July 2023, in addition to the typical Strombolian activity, was characterized by an increase in lava overflows, major explosions and Pyroclastic Density Currents^17,18^. In particular, in the considered period, 23 major explosions occurred, along with at least 30 overflows or lava flows (lasting up to 7 days) and 15 slope and/or rim failures generating Pyroclastic Density Currents. As a consequence, the morphology of both the crater terrace and the steep slopes of the Sciara del Fuoco was profoundly modified, with the generation of collapse scars, canyons (as occurred between October 9^th^ and 15^th^ 2022) and by the accumulation of lava flows and pyroclastic deposits.

Taking into account that the most recent high-resolution surface model of the entire island is dated 2012^19^, and that the volcanic activity occurred between July 2019 and June 2023 generated relevant morphological changes both in the summit area and along the flanks, a dedicated Airborne Lidar survey to update the topography of the entire island was planned. Thanks to this survey, the Digital Surface Model (DSM) of Stromboli Island was updated to August 4^th^ 2023, reproducing both natural and anthropic elements with a spatial resolution of 50 cm.

## Methods

### Airborne Lidar acquisition and DSM generation

**Fig. 2:**
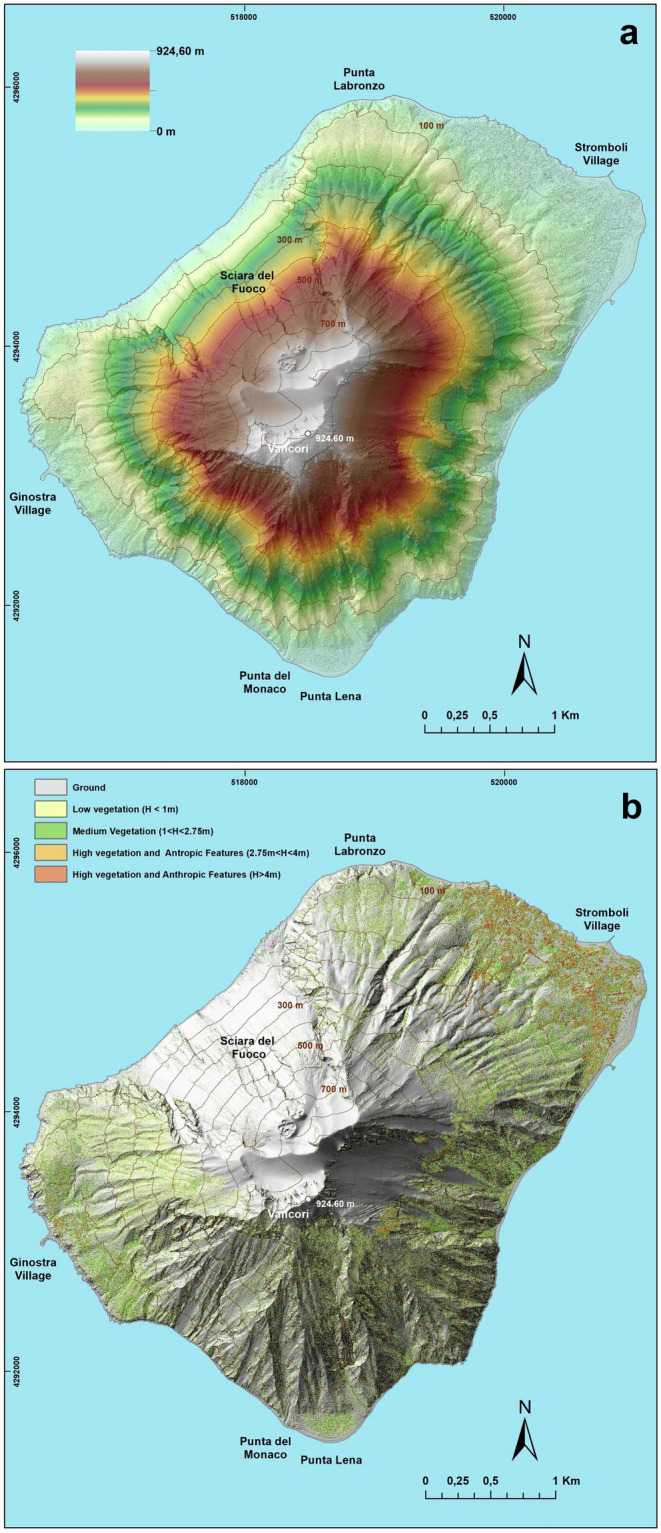
(**a**) Digital Surface Model of Stromboli Island. Elevation data overlaid on the shaded relief image. The contour lines every 10 meters of elevation are visualized in light brown colour. (**b**) Shaded Relief of Stromboli Island. Elevation data are visualised to distinguish ground, vegetation and anthropic elements. The contour lines every 10 meters in height are drawn in light brown colour.

The Lidar survey was carried out on August 4^th^, 2023, flying with a Piper PA 31 aircraft on the entire island of Stromboli, at an average elevation of 2500 m a.g.l. (above ground level), from 8.45 am to 11.13 am (local time). The 3D points acquisition was obtained by using the Leica Geosystems Terrain Mapper System, which integrates the photogrammetric camera Leica RCD30 (image sensor 10,320 × 7,752 pixels) with the Hyperion 2 Lidar sensor. This sensor, operating in the Near Infrared Region (λ = 1.064 μm), and programmed with a scan angle of 36° and a pulse repetition frequency of 900 kHz, acquired a total of 109,018,685 3D points, scanning the surface of the island through five flight lines (Fig. 1). Such planning assured 4 to 6 points per square meter, characterised by an instrumental vertical and horizontal error of ±5 cm (1 σ) and ±13 cm (1 σ), respectively. The points have been correctly geo-localised by using the latest generation Inertial Measurement Unit (IMU), capable of recording global navigation satellite system (GNSS)/Inertial measurements with high precision (https://leica-geosystems.com). The points were acquired in ellipsoid heights and geo-localised in WGS 84 (ETRF2000) UTM N33. Subsequently, the elevations were converted in ortho-metric heights (ITALGEO 2005 geoid), with the ConVERGO and Cartlab 3 software, by using the conversion grids (GK2) provided by Istituto Geografico Militare Italiano (IGMI). In addition to the x, y, and z coordinates, the Lidar sensor recorded a series of returns (in our case, 15 echoes), which allow the classification of different typologies of surfaces hit by the laser beam. The acquired data, stored in 23 LASer format (LAS) files, and containing the information related to geo-localization and return echoes, were elaborated on the TERRASOLID platform (www.terrasolid.com), by using dedicated filtering algorithms^20^ to eliminate outliers and classify the “ground” points from the “not ground” points. Subsequently, these two classes were merged (with a total of 71,298,241 points) and interpolated, to construct a Triangular Irregular Network (TIN) reproducing the elevation variation of the Stromboli surface (including barren soil, vegetation and anthropic features) through a vector model. Then, the vector model was transformed into a raster model (ESRI GRID) at a spatial resolution of 50 cm. The obtained model (Digital Surface Model - DSM) reproduces the topography of the entire island of Stromboli, reaching the maximum elevation (924.60 m a.s.l.) at Vancori (Fig. 2a,b). Figure 2a shows the elevation data of the island surface overlapped to the shaded relief of the DSM, while Fig. 2b distinguishes the different typology of features (ground, vegetation and anthropic), according to the elevation (see legend). Vegetation and anthropic features (“not ground” class) cover 29% of the entire island, except the western sector, dominated by the Sciara del Fuoco. In particular, the vegetation reaches 500 m a.s.l. in the west and north sectors, whereas it extends up 750 m a.s.l. in the southern portion. Anthtropic areas are located, for the most, below 100 m a.s.l., both at Ginostra and Stromboli villages (Fig. 2b). The ground/barren soil affects the entire summit of the volcano (above 700 m a.s.l.), a large portion of the east flank characterized by elevation ≥400 m a.s.l., and the Sciara del Fuoco. These three zones correspond to the areas more frequently affected, directly and/or indirectly, by volcanic activity. In fact, the Sciara del Fuoco is the result of several flank collapses, lava flows and minor lava overflows^21–23^, the summit area morphology is frequently modified due to the presence of the active crater terrace and, finally, the portion of the east flank at an elevation higher than 500 m a.s.l. is often affected by the fall down of ballistic products (lapilli, bombs and spatters), launched during the explosive activity, or by gravity-induced flows, as occurred during the paroxysm of July 3^th^ 2019^12^.

**Fig. 3:**
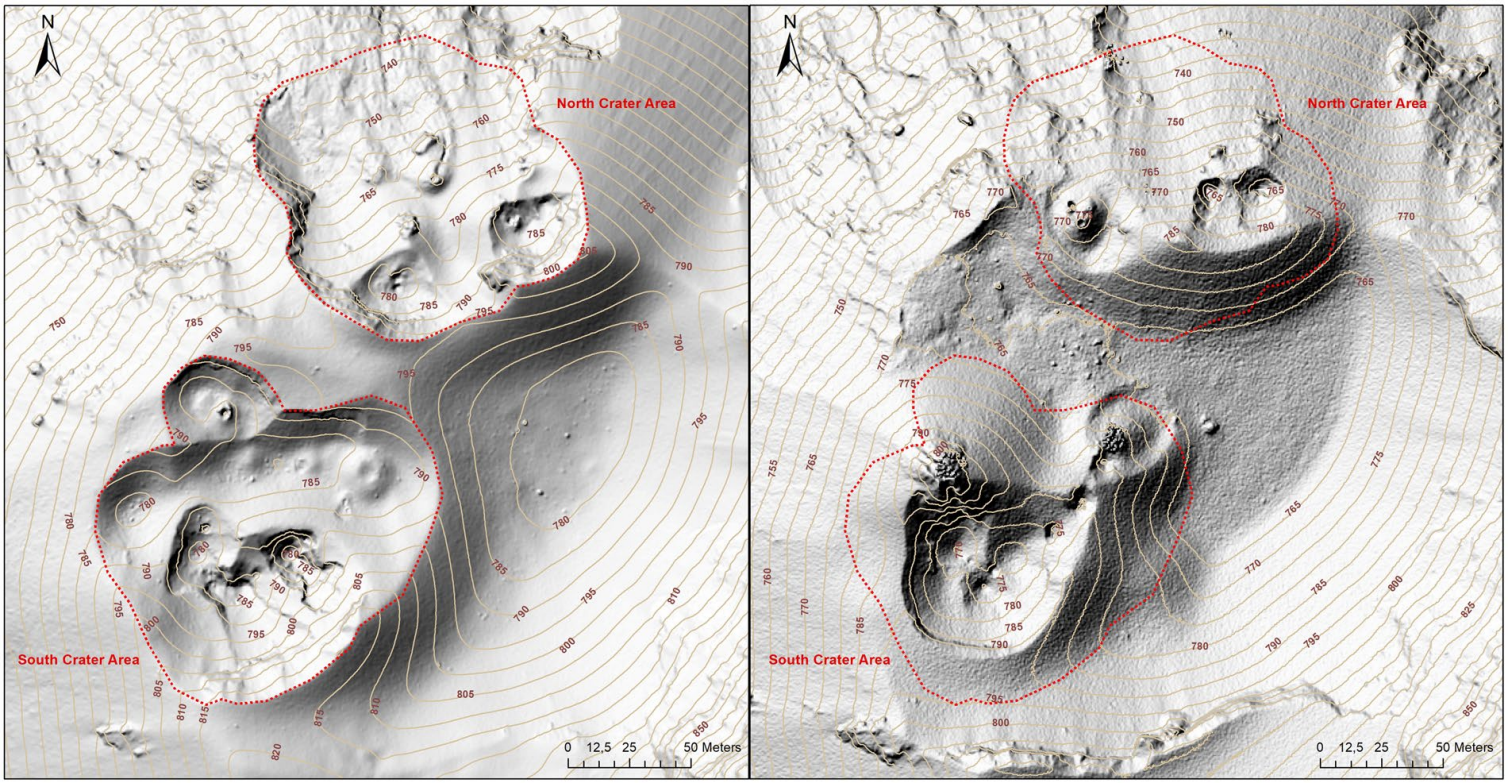
Morphology of the Stromboli summit area dated 2023 (on the left) and 2012 (on the right). The red dotted lines indicate the boundary of the 2023 crater areas.

Focusing on the summit area, the 2023 DSM shows significant morphological changes with respect to the 2012 one, especially in the two crater areas (north and south), as shown in Fig. 3. Overlapping the boundary of the 2023 crater areas on the morphological relief of 2012, the most evident morphological changes result: (i) a relevant enlargement of both crater areas, especially the south one, (ii) the formation and dismantling of volcanic cones within the crater areas, (iii) a significant accumulation (~30 m thickness) of pyroclastic products (mainly ash and lapilli) between the two crater areas.

## Data Records

The data record^24^ consists of a Digital Surface Model at a spatial resolution of 50 cm. The model was obtained by elaborating, on the ESRI platform (ArcGIS Map 10.5), the .las files acquired during the Lidar survey. Details of the Lidar survey are described in the Method section. The Digital Surface Model of Stromboli island is geocoded into WGS 84 (ETRF2000) UTM N33 Coordinate System. It is stored in GeoTIFF file format within the repository 10.13127/stromboli/airborne-lidar-2023. The model is shared under the CC BY 4.0 use license.

## Technical Validation

The validation process of the DSM has been performed by using Ground Control Points (GCPs) measured during two distinct Global Navigation Satellite System - Real Time Kinematics (GNSS-RTK) field campaigns, carried out in July 2024, and retrieved from the available permanent stations of the GNSS monitoring network of Stromboli, managed by INGV-OE. A total of 41 GCPs were acquired, using anthropic and natural features considered stable in space and in time, and well identifiable both in the field and on the Digital Surface Model. The spatial distribution of the GCPs is shown in Fig. 1. It was planned to best cover the entire island, with the exception of inaccessible areas, such as Sciara del Fuoco and several unstable/steep zones.

One field campaign was carried out by using a Stonex S990A receiver, with differential corrections sent by the GNSS Sicilia, while the second one by using a Trimble R6 L1/L2 receiver, with differential corrections from the Spektra Network Real Time Kinematics (NRTK) local area network. Instead, the GNSS monitoring network consists of 4 permanent stations, with 3 of them operating during the Airborne Lidar survey: San Vincenzo station (SVIN), equipped with antenna and receiver Leica GR30/AR20 + LEIM, Punta Lena (SPLN) and Timpone del Fuoco (STDF) stations, equipped with antenna and receiver Leica GR25/AR20. The planimetric coordinates (X,Y) of the GCPs were acquired in WGS 84 (ETRF2000) UTM N33 geodetic cartographic system. The altimetric coordinates (Z), acquired in ellipsoid values, were subsequently transformed into orthometric values by using the ConVERGO software. The coordinates of the 41 GCPs (X,Y, Z) are reported in Table 1, as the site description and the horizontal and vertical errors calculated by the receiver during the acquisitions of each GCP.

**Table 1:** GCPs acquired by GNSS-RTK field campaigns and the permanent stations of the Stromboli GNSS monitoring network.

ID	GCP name	X (m)	Y (m)	Z (m)	Receiver	±XY error (m)	±Z error (m)	Site description
1	S1	520269.25	4295173.23	31.041	Trimble	0.012	0.03	SW corner of the concrete plate
2	S2	520142.60	4295310.54	29.264	Trimble	0.017	0.027	Center of the manhole cover
3	S3	519979.65	4295418.59	29.786	Trimble	0.01	0.015	Center of the manhole cover
4	S4	519886.64	4295470.16	30.781	Trimble	0.011	0.016	Center of the manhole cover
5	S5	519834.94	4295667.17	15.324	Trimble	0.016	0.017	Center of the manhole cover
6	S6	519735.62	4295699.91	9.038	Trimble	0.014	0.018	Center of the manhole cover
7	S8	520550.39	4295300.02	3.305	Trimble	0.022	0.021	Center of the manhole cover
8	S11	520947.22	4295122.22	5.863	Trimble	0.012	0.017	Center of the manhole cover
9	S12	521025.63	4295007.63	3.167	Trimble	0.01	0.014	Center of the manhole cover
10	S13	520896.84	4294679.04	4.268	Trimble	0.013	0.018	Center of the manhole cover
11	S14	520875.50	4294560.09	3.284	Trimble	0.013	0.018	Vertex of the upper right angle between the horizontal and vertical bars of the elipad letter “H”
12	S15	520848.39	4294342.40	2.118	Trimble	0.011	0.016	NE corner of the metallic plate
13	S17	520560.02	4294960.64	35.775	Trimble	0.017	0.021	Center of the manhole cover
14	G1	516446.85	4292969.04	1.758	Trimble	0.012	0.021	NE corner of the metallic plate
15	G2	516465.01	4293002.50	1.996	Trimble	0.014	0.024	SE corner of the drainage grill
16	G3	516564.66	4292980.14	1.786	Trimble	0.013	0.023	Upper benchmark of the ISPRA tide gauge
17	G4	516564.75	4292979.89	1.777	Trimble	0.013	0.022	Lower benchmark of the ISPRA tide gauge
18	G5	516554.48	4293056.88	33.585	Trimble	0.015	0.025	Center of the manhole cover
19	G6	516662.45	4293027.38	56.095	Trimble	0.012	0.037	Vertex of the upper right angle between the horizontal and vertical bars of the elipad letter “H”
20	G12	516795.80	4292955.72	83.255	Trimble	0.015	0.027	Center of the water cistern cover
21	G13	516713.94	4294100.22	100.529	Trimble	0.014	0.02	NE corner of the bench
22	G14	516711.50	4294095.09	100.370	Trimble	0.014	0.021	Metallic pole
23	G15	516635.18	4293451.55	101.339	Trimble	0.015	0.034	Center of the manhole cover
24	G16	516618.17	4293390.61	90.071	Trimble	0.014	0.035	Center of the manhole cover
25	G17	516526.02	4293133.09	31.812	Trimble	0.012	0.045	Center of the painted wind rose
26	PL1	518815.44	4295781.95	109.444	Stonex	0.0066	0.0101	On helipad centre
27	PL2	518809.92	4295788.08	109.378	Stonex	0.0075	0.0114	On the NW corner of the helipad
28	PL5	518830.49	4295787.48	109.203	Stonex	0.005	0.0078	On a manhole near the helipad
29	PM6	518337.94	4291596.18	14.965	Stonex	0.0058	0.0076	On the SE corner of the helipad
30	PM7	518330.55	4291596.91	14.966	Stonex	0.0076	0.0098	On the SW corner of the helipad
31	PM10	518335.41	4291600.24	15.021	Stonex	0.0064	0.0081	On helipad centre
32	SA11	518805.97	4294123.65	794.664	Stonex	0.0066	0.0114	On the corner of a shelter
33	SA12	518809.15	4294121.04	794.646	Stonex	0.0084	0.0148	On the corner of a shelter
34	SA13	518808.45	4294123.70	796.510	Stonex	0.0082	0.0142	On the top of a shelter
35	SA17	518834.98	4293891.48	873.062	Stonex	0.0062	0.0089	On the case of a monitoring station next to the helipad
36	SA18	518851.30	4293914.44	869.493	Stonex	0.0074	0.0107	On the corner of a shelter
37	SA20	518851.65	4293912.17	871.332	Stonex	0.007	0.0098	On the top of a shelter
38	SA21	518844.66	4293908.46	870.708	Stonex	0.0072	0.0098	On the corner of a shelter
39	SPLN	518369.57	4291701.57	42.285	GNSS station	0.005	0.01	GNSS monitoring network
40	STDF	516636.41	4293614.34	139.658	GNSS station	0.005	0.01	GNSS monitoring network
41	SVIN	520334.31	4294920.24	74.173	GNSS station	0.005	0.01	GNSS monitoring network

In the GCP name, the letter before the number indicates the locality where the point was taken (G: Ginostra village, PL: Punta Labronzo, PM: Punta del Monaco, S: Stromboli village, SA: Summit area). The errors in X,Y and Z are calculated by the receiver used during the acquisition and have to be considered both positive and negative as indicated in the header of the table (see the related fields).

The vertical accuracy of the DSM was obtained as the root-mean-square-error (RMSE) of the residuals (Z_L_ − Z_GPS_). Residuals represent the difference in Z between the elevation stored in the DSM cell (Z_L_) that contains the GCP, and the elevation value of the GCP measured on the field (Z_GPS_). If the GCP falls at a distance < 13 cm from the cell boundary, the DSM elevation value (Z_L_) is assumed to be the value stored in the adjacent cell. This distance of 13 cm derives from the instrumental horizontal error of the Lidar sensor at 1 σ. The residuals calculated for the 41 GCPs range from 0.001 m to 0.312 m as shown in Table 2.

**Table 2:** Comparison in elevation between the Lidar and the GCPs.

GCP name	Z GPS (m)	Z LiDAR (m)	Residual (m)	Total Error (m)
PL1	109.444	109.445	0.001	0.130
G3	1.786	1.789	0.003	0.156
PL5	109.203	109.198	0.005	0.126
G4	1.777	1.791	0.014	0.154
G14	100.370	100.388	0.018	0.152
G16	90.071	90.090	0.019	0.180
SPLN	42.285	42.264	0.021	0.130
SA12	794.646	794.624	0.022	0.140
SA11	794.664	794.642	0.022	0.133
S1	31.041	31.074	0.033	0.170
S6	9.038	9.079	0.041	0.146
SA13	796.510	796.558	0.047	0.138
G15	101.339	101.389	0.050	0.178
PM7	14.966	15.019	0.053	0.130
S5	15.324	15.379	0.055	0.144
SA20	871.332	871.272	0.060	0.130
S17	35.775	35.839	0.064	0.152
S15	2.118	2.182	0.064	0.142
SA21	870.708	870.642	0.066	0.130
SA17	873.062	873.132	0.070	0.128
STDF	139.658	139.579	0.079	0.130
G1	1.758	1.838	0.080	0.152
S14	3.284	3.369	0.085	0.146
PL2	109.378	109.292	0.086	0.133
PM6	14.965	15.052	0.087	0.125
G2	1.996	2.093	0.097	0.158
S13	4.268	4.371	0.103	0.146
G17	31.812	31.918	0.106	0.200
PM10	15.021	15.129	0.108	0.126
G12	83.255	83.147	0.108	0.164
G5	33.585	33.695	0.110	0.160
SVIN	74.173	74.056	0.117	0.130
G6	56.095	56.216	0.121	0.184
S4	30.781	30.913	0.132	0.142
S3	29.786	29.921	0.135	0.140
S2	29.264	29.409	0.145	0.164
G13	100.529	100.676	0.147	0.150
S12	3.167	3.341	0.174	0.138
SA18	869.493	869.302	0.191	0.131
S8	3.305	3.514	0.209	0.152
S11	5.863	6.175	0.312	0.144

For each GCP, the residual is expressed as a module of Z_L_ − Z_GPS_ and the Total Error is obtained considering the entire range of each treated error.

**Fig. 4:**
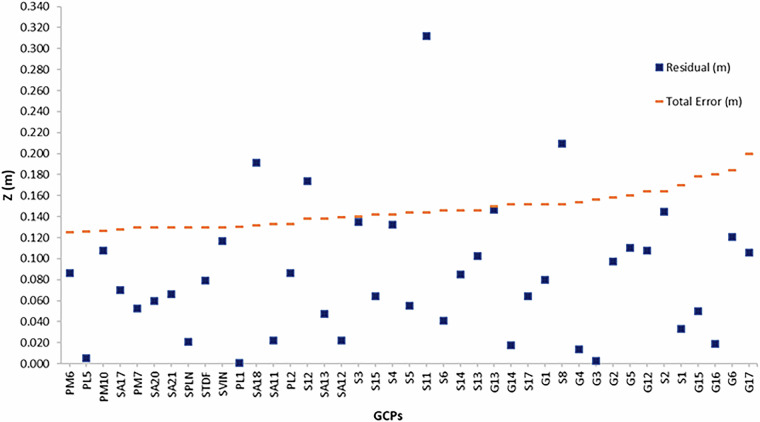
Residuals (Z_L_ − Z_GPS_) between DSM and GPS elevations and the sum of the vertical instrumental errors (Total Error) referred to each GCP (Table 1). The Y axis reports the value of the Total Error and Residuals expressed in meters (Z).

The vertical accuracy of the DSM results in 8 cm and such value is consistent with the instrumental vertical error (±5 cm) of the Lidar sensor used in the acquisition survey. In addition, to evaluate the reliability of the obtained accuracy, the Residual and the Total Error (Table 2) were compared for each GCP. In detail, the Total Error is calculated as the sum of the instrumental errors of GPS, Lidar and the uncertainty of ±0.5 cm referred to field measurements. The DSM is considered validated when the Residual is minor than the Total Error. This is true for 90% of the acquired GCPs, as shown in the plot of Fig. 4, confirming the high quality of the DSM.

## Data Availability

No custom code was used to generate or process the data described in the manuscript.
